# Editorial: Bridging the knowledge gap: mental health, substance use disorders, and mortality in women

**DOI:** 10.3389/fgwh.2025.1721670

**Published:** 2025-11-21

**Authors:** Amar D. Mandavia, Leah C. Susser, Sohye Kim

**Affiliations:** 1 National Center for PTSD, Behavioral Science Division, Boston, MA, United States; 2 Department of Psychiatry, Chobanian & Avedisian School of Medicine, Boston University, Boston, MA, United States; 3 Department of Psychiatry, Weill Cornell Medicine, New York, NY, United States; 4 Eunice Kennedy Shriver Center, University of Massachusetts Chan Medical School, Worcester, MA, United States; 5 Departments of Psychiatry, Pediatrics, and Obstetrics & Gynecology, University of Massachusetts Chan Medical School, Worcester, MA, United States

**Keywords:** womens health, social detereminants, mortality, substance use disorder, mental illness & social determinants

Mental health conditions and substance use disorders (SUDs) among women represent one of the most pressing global health challenges of our time. Women are nearly twice as likely as men to experience major depression (1) and have significantly higher lifetime risk of anxiety disorders (2, 3). Trauma-related disorders, including PTSD, also disproportionately affect women, often in the context of sex-based violence and adverse social determinants (2). Women with SUD face unique biological vulnerabilities and social barriers, developing complications more rapidly than men and experiencing stigma, caregiving burdens, and treatment access inequities (4–6). Despite this elevated burden, research and interventions have too often neglected women's specific biological, social, and cultural contexts. Historically, women—particularly those who are pregnant, postpartum, or from marginalized groups—have been excluded from clinical research, leaving critical gaps in understanding mechanisms, risk factors, and effective interventions (7, 8).

This research topic brings together a diverse set of contributions addressing these knowledge gaps. These articles advance our understanding of how women's mental health and SUDs intersect with morbidity and mortality, while highlighting innovative solutions ranging from community-driven interventions to health systems integration and big-data approaches.

## Expanding conceptual frameworks for women's mental health

Friedhoff and colleagues underscore the importance of integrating the concept of *matrescence*—the developmental transition into motherhood—into perinatal psychiatry (Friedhoff et al.). They highlight how maternal mental health cannot be reduced to pathology alone; instead, it should be understood as a holistic transformation encompassing biological, social, cultural, and existential domains. This perspective challenges existing nosology, which often stigmatizes perinatal women while failing to capture their lived experiences. Their call echoes a broader theme of this issue: the need to center women's voices in shaping mental healthcare systems.

Skommer and Gunesh extend this agenda by examining the intersection of autism spectrum disorder (ASD), menstruation, and mental health (Skommer and Gunesh). Their scoping review reveals how women and gender-diverse individuals with ASD face unique vulnerabilities at reproductive transitions such as menarche and menopause—periods often overlooked in both ASD and women's health research. By framing menstruation within a biopsychosocial model, they highlight structural neglect and call for inclusive, sex-sensitive approaches that link neurodevelopmental and reproductive health.

## Biological, social, and structural determinants of risk

A core focus of this issue is understanding how structural inequities amplify women's risk for poor health outcomes. Martinez-Gonzalez and Santiago document the stark disparities in access to evidence-based treatment for opioid use disorder (OUD) among Hispanic pregnant individuals (Martinez-Gonzalez and Santiago). Despite the safety and efficacy of medications for OUD, systemic inequities in prescribing practices and retention place this population at elevated risk for overdose, maternal mortality, and adverse neonatal outcomes. Their perspective highlights the urgency of culturally tailored interventions that dismantle stigma, expand harm reduction, and integrate perinatal addiction care into mainstream health systems.

**Figure 1:**
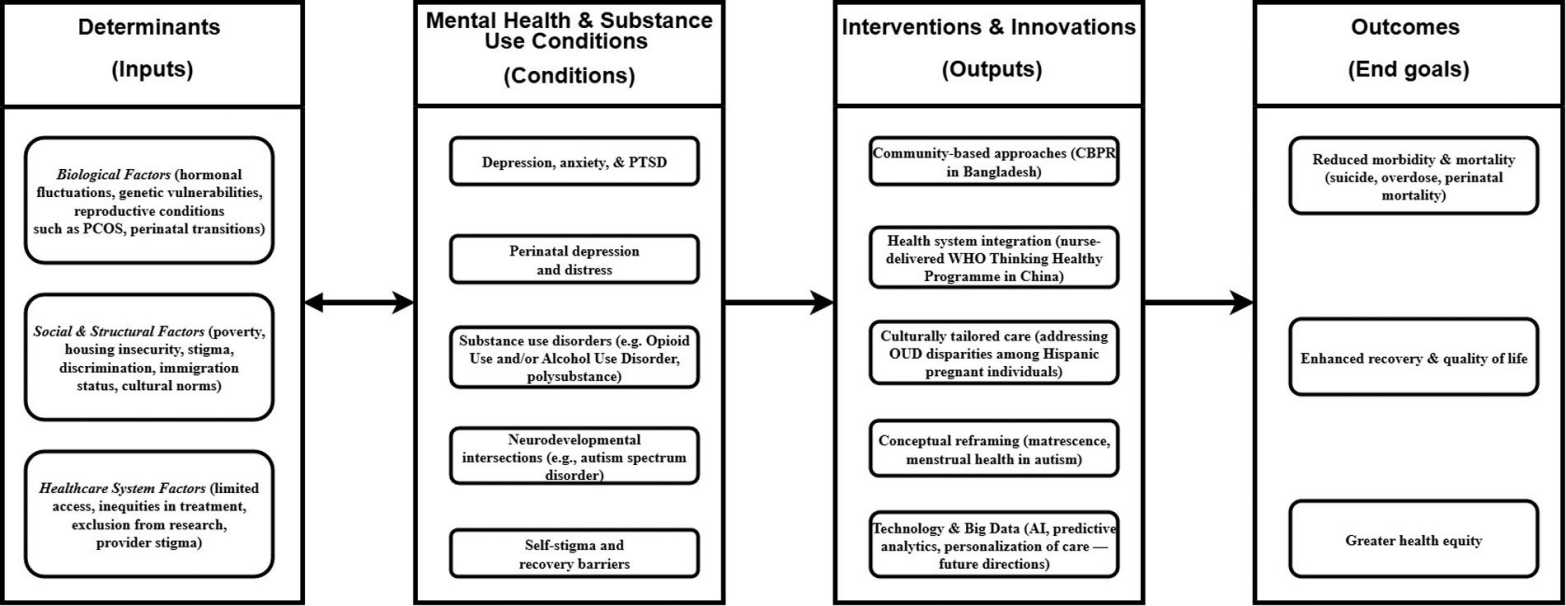
Conceptual framework linking determinants, mental health/SUD conditions, interventions, and outcomes for women. Inputs (biological, social, and healthcare system determinants) contribute to the development and exacerbation of conditions (i.e., mental health and substance use). Outputs (interventions such as community-based care, health system integration, and conceptual reframing) lead to improved end goals: reduced morbidity and mortality, enhanced recovery, and greater health equity.

Yang and colleagues add further nuance by identifying psychosocial and biological contributors to mental health comorbidities in women with polycystic ovary syndrome (PCOS; Yang et al.). In a large clinical sample, they report alarmingly high rates of depression (47.7%) and anxiety (39.9%) in women with PCOS. The authors propose that these high rates may in part be driven by sleep disruptions and daytime dysfunction. These findings highlight a potential bidirectional pathway between reproductive endocrinology and mental health, emphasizing the need for comprehensive screening and integrated treatment strategies. Figure 1 illustrates a conceptual framework mapping how biological, social, and healthcare determinants (“Inputs”) shape women's mental health and substance use conditions (“Conditions”), and how interventions (“Outputs”) translate into improved health and equity (“End goals”).

## Innovation in interventions

Two contributions demonstrate how innovative delivery models can expand access to effective interventions. Nisar and colleagues piloted integration of the World Health Organization's *Thinking Healthy Programme* [a cognitive-behavioral therapy (CBT)–based intervention for perinatal depression] into routine antenatal care in China (Nisaret al.). Delivered by nurses within hospital-based pregnancy schools, authors found that the intervention was feasible, acceptable, and associated with reductions in depressive and anxiety symptoms. Their work exemplifies task-shifting and health system integration as scalable approaches to closing the massive treatment gap for perinatal depression globally.

Complementing this, Afreen and colleagues showcase the promise of *community-based participatory research* (CBPR) in rural Bangladesh (Afreen et al.). By engaging women, community health workers, and local technology hubs, they developed strategies to improve mental health literacy and challenge stigmatization. Their model not only empowers women to advocate for their health but also leverages local infrastructure for sustainable solutions—illustrating the power of bottom-up approaches in resource-limited settings.

## Stigma, recovery, and sex differences

Finally, Leon-Morales and colleagues work on “self-stigma”, sex, and personal recovery in psychotic spectrum disorders extends our understanding of how sex mediates recovery pathways. The authors report that, for women, greater “self-stigma” is associated with diminished hope, confidence, and symptom control, whereas in men, alienation paradoxically correlated with higher orientation toward success. These sex-differentiated findings underscore the need for recovery-oriented interventions that are sensitive to sex-based experiences of stigma.

## Toward a future of sex-sensitive, inclusive, and data-driven care

Across these diverse contributions, several unifying themes emerge: (a) Centering Women's Lived Experience: Whether through matrescence, menstrual health, or stigma in psychosis, these studies call for theoretical and clinical frameworks that reflect women's lived experiences and realities rather than pathologize them; (b) Addressing Inequities: From rural Bangladesh to Hispanic perinatal populations in the U.S., disparities in access to care remain profound, requiring structural reforms and culturally responsive models; (c) Integrating Biological and Psychosocial Determinants: Research on PCOS and perinatal depression demonstrates how reproductive biology, sleep, and social context interact to shape morbidity and mortality risks; (d) Scaling Effective Interventions: Nurse-delivered CBT and CBPR-based literacy initiatives illustrate feasible, sustainable models for addressing the treatment gap in both high- and low-resource settings; (e) Harnessing Innovation: Future directions must leverage big data, machine learning, and interdisciplinary collaboration to personalize interventions and identify modifiable risk factors at scale.

Together, the contributions in this issue expand the frontiers of women's mental health research and care. They chart a path toward a future where interventions are not only evidence-based but also sex-sensitive, culturally grounded, and equitably delivered. Addressing women's mental health and substance use is not peripheral to global health—it is central to reducing morbidity and mortality and to advancing health equity worldwide.
